# Perlecan: An Islet Basement Membrane Protein with Protective Anti-Inflammatory Characteristics

**DOI:** 10.3390/bioengineering11080828

**Published:** 2024-08-13

**Authors:** Daniel Brandhorst, Heide Brandhorst, Samuel Acreman, Paul R. V. Johnson

**Affiliations:** 1 Islet Transplant Research Group, Nuffield Department of Surgical Sciences, University of Oxford, Oxford OX3 9DU, UK; heide.brandhorst@nds.ox.ac.uk (H.B.);; 2 Oxford Consortium for Islet Transplantation, Oxford Centre for Diabetes, Endocrinology, and Metabolism (OCDEM), Churchill Hospital, University of Oxford, Oxford OX3 7LE, UK

**Keywords:** collagen type IV, extracellular matrix proteins, human islet isolation, hypoxia, inflammation, islet basement membrane, laminin-521, perlecan

## Abstract

Throughout the isolation process, human islets are subjected to destruction of the islet basement membrane (BM) and reduced oxygen supply. Reconstruction of the BM represents an option to improve islet function and survival post-transplant and may particularly be relevant for islet encapsulation devices and scaffolds. In the present study, we assessed whether Perlecan, used alone or combined with the BM proteins (BMPs) Collagen-IV and Laminin-521, has the ability to protect isolated human islets from hypoxia-induced damage. Islets isolated from the pancreas of seven different organ donors were cultured for 4–5 days at 2% oxygen in plain CMRL (sham-treated controls) or in CMRL supplemented with BMPs used either alone or in combination. Postculture, islets were characterized regarding survival, in vitro function and production of chemokines and reactive oxygen species (ROS). Individually added BMPs significantly doubled islet survival and increased in vitro function. Combining BMPs did not provide a synergistic effect. Among the tested BMPs, Perlecan demonstrated the significantly strongest inhibitory effect on chemokine and ROS production when compared with sham-treatment (*p* < 0.001). Perlecan may be useful to improve islet survival prior to and after transplantation. Its anti-inflammatory potency should be considered to optimise encapsulation and scaffolds to protect isolated human islets post-transplant.

## 1. Introduction

Over the last couple of decades, clinical islet transplantation has achieved excellent results for specific indications for patients with type 1 diabetes mellitus [1]. Human islet isolation from organ donor pancreata is a key step in islet transplantation which requires the dissociation of the pancreas using a combination of enzymatic digestion and mechanical agitation. The critical step within this complex procedure is to release islets from within the surrounding pancreatic acinar tissue by dispersing the extracellular matrix (ECM). This involves enzymatic cleavage of the islet basement membrane (BM) [2,3]. Islet BMs are formed by large supra-structures mainly composed of Collagen-IV (COL-4) and Laminin-511 (L-511) which are linked and assembled through Nidogen-1 (NID-1) and Perlecan (PLC) [4,5,6,7,8,9]. Since islet BM proteins (BMPs) are essential for the transduction of pro-survival signals between the ECM and the subcellular structures of islet cells via islet-expressed integrins [10,11,12,13,14], the interruption of integrin-mediated communication induces the activation of pro-inflammatory and pro-apoptotic pathways. This results in the loss of the morphological and functional integrity of isolated islets after isolation and during culture [11,15,16,17,18,19,20,21]. These findings clearly underline the need for strategies to embed isolated islets into a microenvironment that improves the functional and morphological survival of islets prior to and after transplantation. This may be particularly relevant for islets placed in immuno-isolating devices or scaffolds as previous studies have demonstrated that BMPs can contribute to a biocompatible microenvironment that enhances the survival and function of microencapsulated islets [22,23,24].

Because COL-IV and L-511 or L-521 represent the major components of BMs [6,14,25,26,27,28,29,30], the majority of islet studies have investigated the effect of these BMPs on function and survival of isolated and cultured islets [23,24,31,32,33,34,35]. In the present study, PLC, a member of the heparan sulphate proteoglycan (HSPG) group, was used as integrative linker molecule for COL-4 and L-521. Apart from its role as a biomechanical stabilizer, numerous other vital functions of PLC have been described. Amongst those, the binding of growth factors, such as VEGF and its signalling, as well as the regulation of angiogenesis and growth of several tissues underline the relevance of this ubiquitous molecule for normal development and physiology [36,37]. The aim of the present attempt was to evaluate the protective efficiency of PLC for isolated human islets exposed to a pro-inflammatory environment which is initially induced during the islet isolation procedure and is also present after transplantation.

## 2. Materials and Methods

### 2.1. Human Islet Isolation

All donor pancreata were voluntarily donated with written consent according to the Declaration of Istanbul. Ethical approval for using isolated human islets for research was given by the Human Tissue Authority (22496) and by the NHS National Research Ethics Service (10/H0605/41). Pancreata were retrieved from 7 (2 female/5 male) human multi-organ donors with brain death with a mean age of 47.3 ± 3.1 years (±standard error) and a mean body mass index of 27.0 ± 2.5 kg/m^2^. All pancreata were preserved with University of Wisconsin solution (Bridge to Life, London, UK) for a mean cold ischaemia time of 4.8 ± 0.5 h. Human islets were isolated and purified using standard isolation techniques as previously described in detail [3].

**Figure 1:**
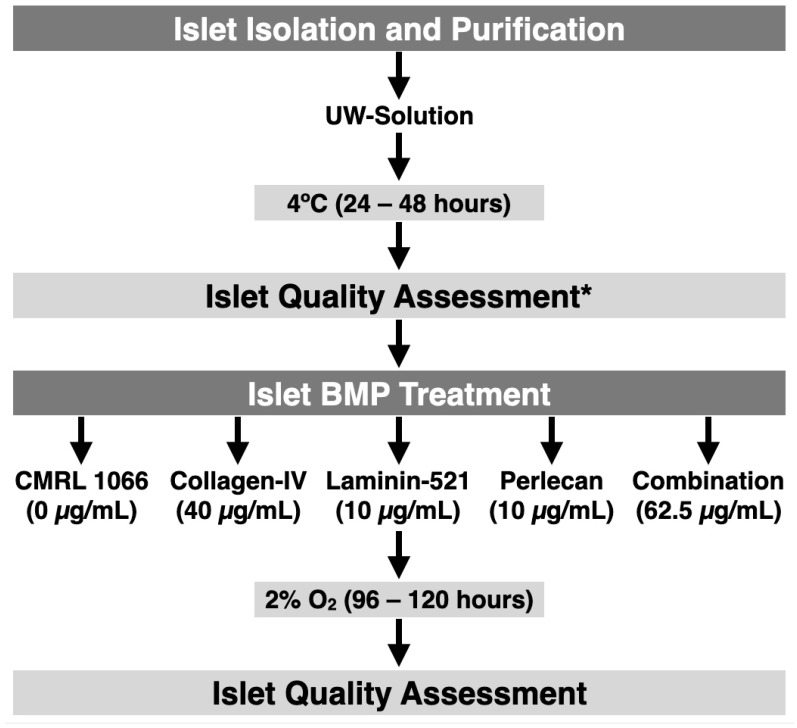
Experimental design of the study. * The first islet quality assessment, taking place before starting the treatment, did not include the glucose-stimulated insulin secretion as performed after 4–5 days of treatment.

The experimental design of the study is shown in Figure 1.

### 2.2. Human Islet Culture

After isolation and purification, a maximum of 550 islet equivalents (IEQs) were placed per well in 24-well plates (Greiner Bio-One, Stonehouse, UK) and suspended in 500 μL of the culture medium CMRL 1066, respectively. This corresponds to a seeding density of 250 IEQ per cm^2^ on average. Culture medium was supplemented with 20 mmol/L HEPES, 2 mmol/L L-glutamine, 200 units/mL penicillin, 200 μg/mL streptomycin (all reagents from Life Technologies, Paisley, UK) and 2% foetal calf serum (PAA Laboratories, Pasching, Austria). Isolated islets were cultured for four to five days in a hypoxic atmosphere (2% oxygen, 5% carbon dioxide) at 37 °C in the presence or absence of 40 μg/mL Collagen-IV (COL-4, Sigma-Aldrich, Dorset, UK), 10 μg/mL Laminin-521 (L-521, Biolam-ina, Stockholm, Sweden) or 10 μg/mL Perlecan (PLC, USCN Life Science, Brussels, Belgium) used either alone or in combination by adding 40 μg/mL COL-4 plus 10 μg/mL L-521 plus 10 μg/mL PLC, i.e., using a total amount of 60 μg/mL BMPs. Although L-511 is the only Laminin isoform present in the human islet BM [38], we could demonstrate in a previous study that L-511 can be replaced by L-521 without experiencing a lower efficiency or any disadvantages [35].

### 2.3. Islet Characterisation

Before and after culture, samples of defined volume were collected from any treatment group and stained with dithizone (Sigma-Aldrich) to visually determine the number of actual islets defined as dithizone-positive size-independent insulin-containing cell clusters. Any counted islet was categorised according to its size and mathematically converted into islet equivalents (IEQs) considering the individual volume of counted islets as previously described in detail [39]. This conversion was normalized to a “standard” islet of 150 μm of diameter which is defined as one IEQ. Proportional IEQ yield (%) was normalized to IEQ yield as counted after sham-treatment. Islet morphological integrity was determined by calculating the islet disintegration index dividing the number of size-independent actual islets (IN) by IEQs (islet disintegration index = IN ÷ IEQ) [40]. Islet viability was assessed utilising 0.67 μmol/L of fluorescein diacetate (FDA, Sigma-Aldrich) and 4.0 μmol/L of propidium iodide (PI, Sigma-Aldrich) for staining of viable and dead cells, respectively [41]. The fluorescence intensity (FI) of FDA-PI was quantified utilising a fluorometric plate reader as previously described [42]. IEQ overall survival was calculated considering the recovery of viable cells only, stained exclusively by FDA and not being penetrated by PI.

In vitro function of 20 hand-picked islets of similar size (150–200 μm) was assessed in duplicate during static glucose incubation. These islets were seeded on 8 μm-pore size filter inserts, transferred into 24-well plates and sequentially incubated for 45 min in 1 mL of Krebs–Ringer buffer supplemented with 2.0 mmol/L glucose followed by 45 min at 20 mmol/L glucose and followed by a second period of 45 min at 2 mmol/L glucose. After stimulation, islets were recovered and sonified in 1 mL of distilled water. An aliquot of the disintegrated islet cell suspension was mixed with acid ethanol at a ratio of 1:4 followed by overnight insulin extraction at 4 °C [43]. Before performance of the human insulin-specific enzyme immunoassay (Mercodia, Uppsala, Sweden), samples were diluted and neutralized by Krebs–Ringer buffer. The glucose stimulation index (GSI) was calculated by dividing the insulin release at 20 mmol/L glucose by the mean of the two basal periods.

Production of reactive oxygen species (ROS) was determined by measuring the intra-islet conversion of dichlorofluorescein diacetate into fluorescent dichlorodihydrofluorescein as previously described in detail [44]. After culture in a hypoxic atmosphere, islet-preconditioned supernatants were collected and assessed for secretion of hypoxia- and inflammation-related chemokines. Release of Interleukin-1 beta (IL-1β), IL-6, IL-8, monocyte chemoattractant protein-1 (MCP-1), tumour necrosis factor alpha (TNF-α) and vascular endothelial growth factor A (VEGF-A) was detected utilising enzyme immunoassays specific for human chemokines (Abcam, Cambridge, UK). Early apoptosis was demonstrated by exclusive staining of phospatidylserine using Annexin-V. In contrast, islet late apoptosis was determined by simultaneous staining with Annexin-V FITC (Becton-Dickinson Biosciences, Oxford, UK) as well as PI used at a concentration of 450 ng/mL and 4.0 μmol/L, respectively [45,46].

ROS production as well as chemokine release, glucose-stimulated insulin secretion, necrosis and expression of apoptosis markers were normalized to IEQs.

### 2.4. Statistical Analysis

Statistical analysis and graphical presentations were performed using Prism 9.5.1 (GraphPad, La Jolla, CA, USA). Analysis of data was carried out using the nonparametric Friedman test followed by Dunn’s test for multiple comparisons or by the Wilcoxon test for subsequent insulin release at 2 and 20 mmol/L of glucose. Correlation analysis was performed calculating the nonparametric Spearman’s correlation coefficient (r) subsequent to outlier identification using the ROUT method at a Q-level of 1% [47]. Where appropriate, data were normalized to sham treatment. Differences were considered significant at *p* less than 0.05. *p*-values larger than 0.05 were termed nonsignificant (NS). Results are generally expressed as mean ± standard error (SEM).

## 3. Results

### 3.1. Protective Effect of Islet BMPs on Islet Survival and In Vitro Function

**Figure 2:**
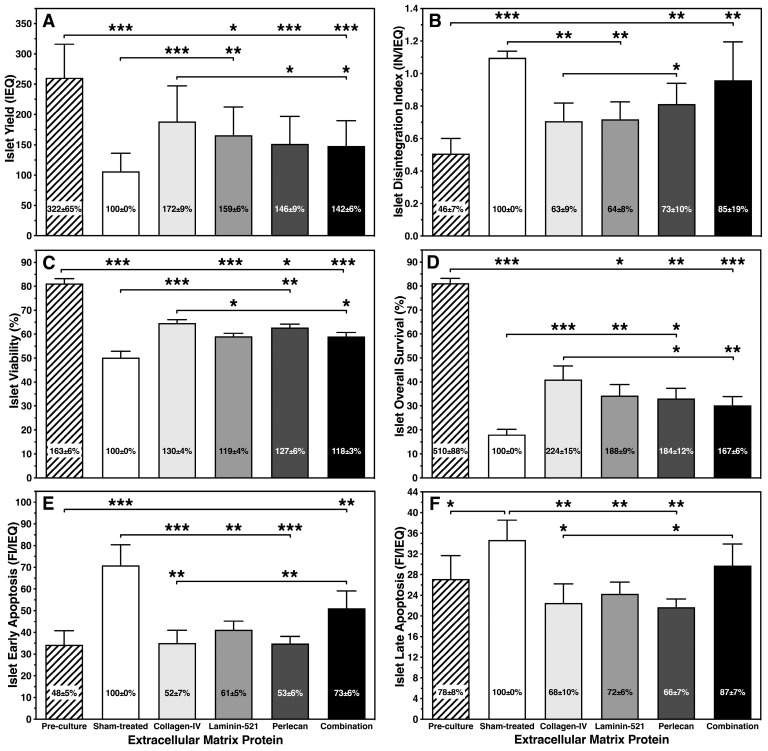
Effect of different islet BMPs on (**A**) islet yield, (**B**) islet disintegration index, (**C**) islet viability, (**D**) islet overall survival, (**E**) islet early apoptosis and (**F**) islet late apoptosis after 4–5 days of culture in hypoxia (n = 7). Lines/arrows indicate *** *p* < 0.001, ** *p* < 0.01, * *p* < 0.05 for comparison of experimental groups. Figures inside bars display variable figures normalized to sham-treated islets.

As shown in Figure 2A, exposure of isolated human islets to a hypoxic atmosphere reduced the number of initially incubated islets substantially. Apart from the islets treated with COL-4 (NS), the yield of IEQ dropped significantly during four to five days of hypoxic culture at 2% oxygen in all experimental groups when compared with the pre-culture islets. When opposed to sham-treated islets a significantly improved recovery of IEQ could be obtained after treatment with COL-4 (*p* < 0.001) and L-521 (*p* < 0.01) but not with PLC (NS) or with the combined BMPs (NS). IEQ loss was associated with increased morphological fragmentation as expressed by the disintegration index which was highest in sham-treated islets (*p* < 0.001 vs. pre-culture) but was also significant in the PLC group (*p* < 0.01) and the combination group (*p* < 0.01) (Figure 2B). In comparison with sham-treated islets, only COL-4 (*p* < 0.01) and L-521 (*p* < 0.01) protected islets from hypoxia-induced disintegration. Comparing the outcomes of all treatment groups, COL-4 seemed to have the strongest protective capacity for IEQ yield and corresponding morphological integrity as expressed by the disintegration index (*p* < 0.05 vs. PLC, combination).

**Figure 3:**
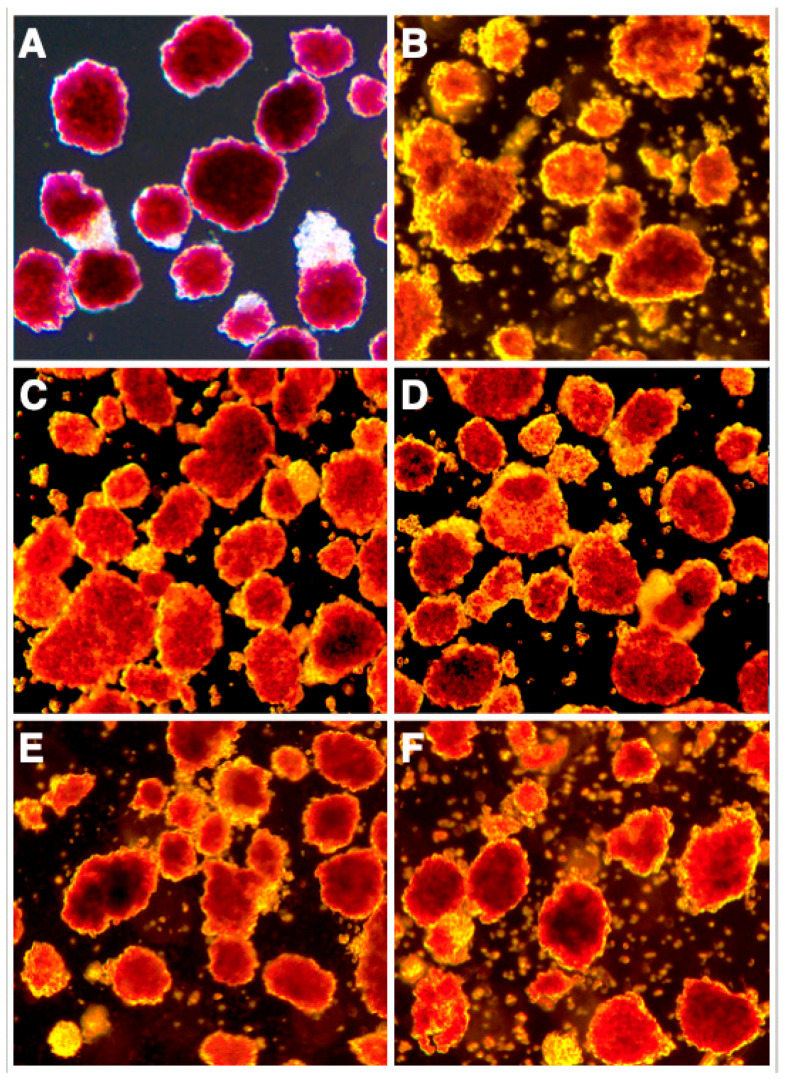
Effect of different islet BMPs on the morphology of dithizone-stained human islets after 4–5 days of culture in hypoxia. Treatment groups are (**A**) pre-culture, (**B**) sham-treatment, (**C**) COL-4, (**D**) L-521, (**E**) PLC and (**F**) the combination of COL-4 plus L-521 plus PLC. All treatment groups were isolated from the same donor pancreas (original magnification × 50).

Islet morphological integrity and disintegration is also demonstrated by light microscopy in Figure 3. Whilst dithizone-stained islets pre-culture showed an ovoid shape with a clearly defined periphery (Figure 3A), sham-treated islets (Figure 3B) and islets treated for 4–5 days with the combination (Figure 3F) are characterized by an irregular and disintegrated periphery accompanied by an accumulation of single cells at the bottom of the wells. In contrast, islets treated with COL-4 (Figure 3C), or L-521 (Figure 3D) still had a well-preserved periphery and were underlaid with only a few dropped single cells.

The differences in membrane integrity as measured by the FDA-PI viability assay were relatively small between the different experimental groups with the exception of sham-treated islets which lost nearly 40% of the initial viability (*p* < 0.001 vs. pre-culture; Figure 2C). Compared with L-521 and the combination, COL-4-treated islets showed a significantly higher viability (*p* < 0.05). When IEQ overall survival was calculated, i.e., the recovery of viable cells only, simultaneously excluding the recovery of dead cells, it became obvious that treatment of islets with individually used BMPs nearly doubled the IEQ overall survival after hypoxic culture in comparison with sham-treated islets (*p* < 0.001 vs. COL-4; *p* < 0.01 vs. L-521; *p* < 0.05 vs. PLC; NS vs. combination; Figure 2D). As observed for islet yield, COL-4 demonstrated the highest potency to preserve IEQ overall survival when compared with PLC (*p* < 0.05) and the combination (*p* < 0.01).

**Figure 4:**
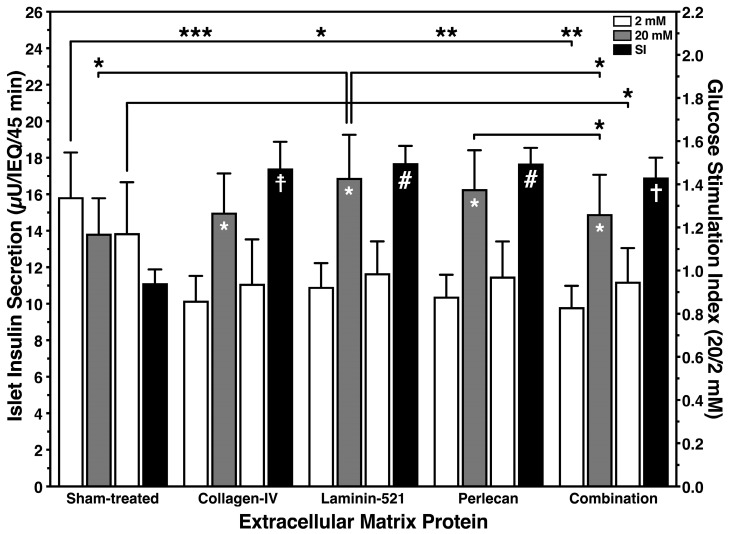
Glucose-stimulated insulin secretion after 4–5 days of culture in a hypoxic atmosphere utilising different islet BMPs as supplements for culture media. Basal (white bars), stimulated insulin release (grey bars) and GSI (black bars) of 20 human islets are normalized to IEQ. Symbols inside white and grey bars indicate * *p* < 0.05 for 2 vs. 20 mmol/L of glucose. Lines/arrows indicate *** *p* < 0.001, ** *p* < 0.01, * *p* < 0.05 for comparison of basal or stimulated insulin release. Symbols inside back bars indicate # *p* < 0.001, ‡ *p* < 0.01, † *p* < 0.05 for comparison of GSI calculated for different treatment groups.

Apart from their morphological integrity, the functional capacity of cultured islets was significantly affected by the hypoxic atmosphere during culture as well. As detailed in Figure 4, hypoxic sham-treated islets were not able to properly respond to an increase of the glucose concentration. This finding might be related to the fact that the initial basal insulin release of sham-treated islets was significantly higher in comparison with the other treatment groups (*p* < 0.001 vs. COL-4; *p* < 0.05 vs. L-521; *p* < 0.01 vs. PLC, combination). In addition, sham-treated islets could not downregulate insulin release when glucose was switched back from a stimulatory to a basal level. In contrast, when islet BMPs had been present during hypoxic culture a physiological insulin response could be observed in BMP-treated islets. Consequently, the GSI, reflecting the secretory capacity of islets, was similar in all treatment groups except in sham-treated islets which showed a significantly reduced GSI (*p* < 0.01 vs. COL-4; *p* < 0.001 vs. L-521, PLC; *p* < 0.05 vs. combination; Figure 4).

### 3.2. Inhibitory Effect of Islet BMPs on Islet-Related Inflammation and Apoptosis

**Figure 5:**
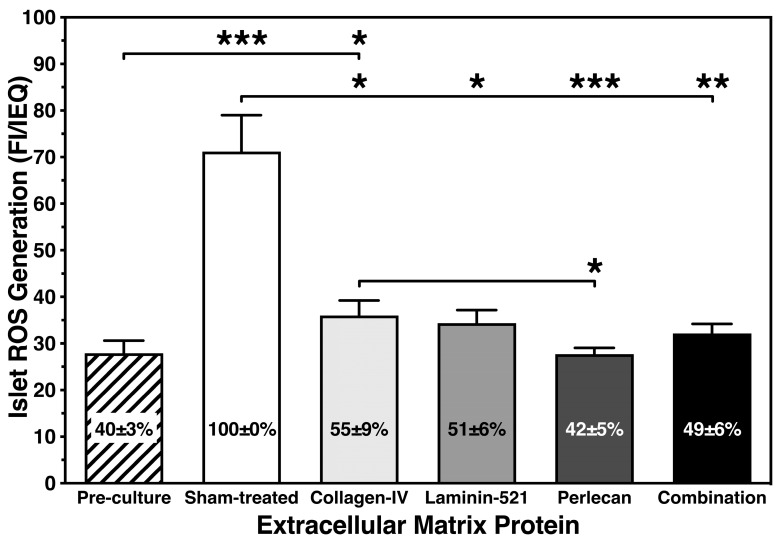
Effect of different islet BMPs on islet generation of ROS normalized to IEQ after 4–5 days of culture in hypoxia (n = 7). Lines/arrows indicate *** *p* < 0.001, ** *p* < 0.01, * *p* < 0.05 for comparison of experimental groups. Figures inside bars display chemokine release normalized to sham-treated islets.

**Figure 6:**
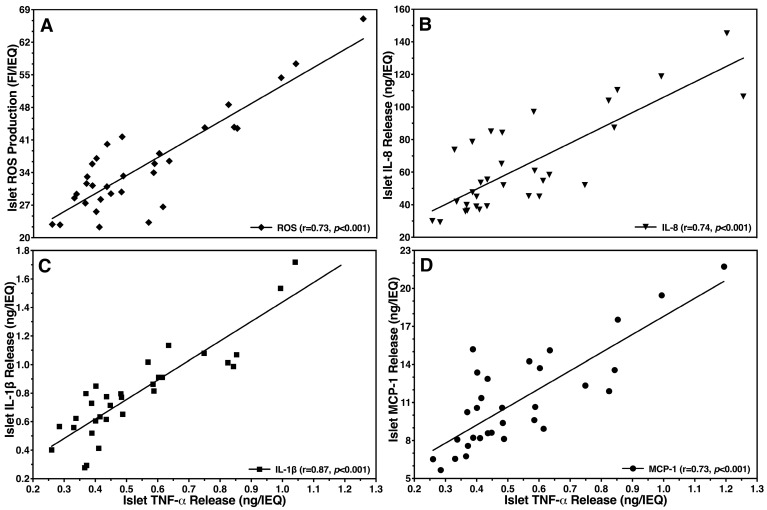
Effect of islet TNF-α release in hypoxic atmosphere on (**A**) intra-islet production of ROS (◆) or release of (**B**) IL-8 (▼), (**C**) IL-1β (■) and (**D**) MCP-1 (●). Each data point represents the release or, respectively, production of different chemokines or ROS in relation to the corresponding TNF-α secretion. The correlation coefficient (r) was calculated using Spearman’s rank correlation after outlier identification (n = 31).

As shown in Figure 5, several days of culture in hypoxia had a significant effect on the production of ROS. In sham-treated islets, the intra-islet formation of ROS increased by more than two-fold when compared with islets preculture. In contrast, the presence of islet BMPs approximately halved the ROS generation in sham-treated islets. PCL was most effective to reduce the production of ROS in comparison with sham-treated islets (Figure 5). Unexpectedly, we could not verify a significantly harmful effect of ROS on glucose-stimulated insulin release (r = −0.16, NS) or on early apoptosis (r = 0.32, NS) of hypoxic islets utilizing Spearman’s rank correlation. On the other hand, a close association of ROS production with chemokine release was confirmed by the tight correlation that was found between ROS and TNF-α (r = 0.73, *p* < 0.001; Figure 6A).

**Figure 7:**
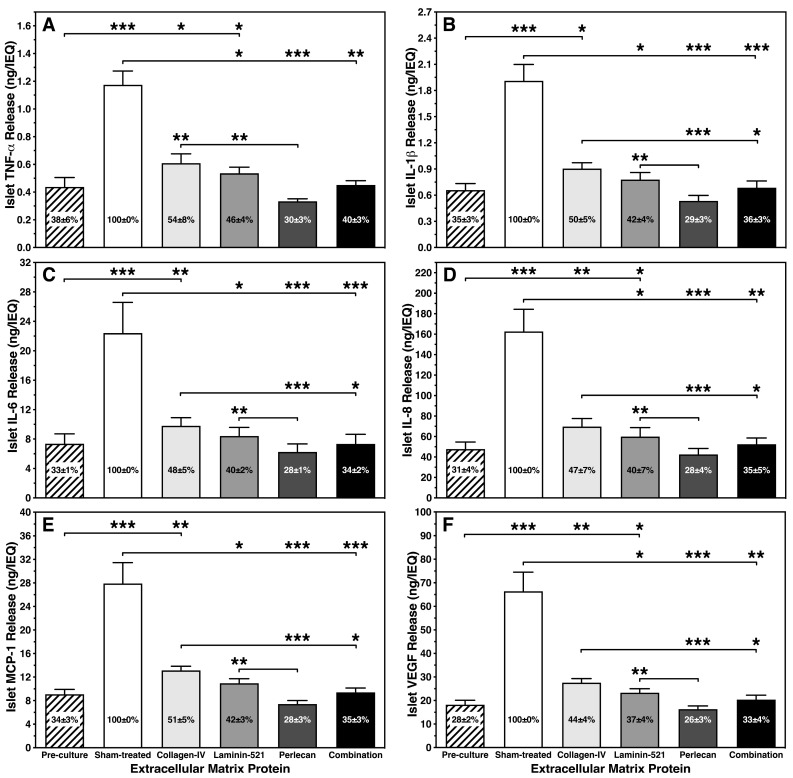
Effect of different islet BMPs on release of (**A**) TNF-α, (**B**) IL-1β, (**C**) IL-6, (**D**) IL-8, (**E**) MCP-1 and (**F**) VEGF normalized to IEQs after 4–5 days of culture in hypoxia (n = 7). Lines/arrows indicate *** *p* < 0.001, ** *p* < 0.01, * *p* < 0.05 for comparison of experimental groups. Figures inside bars display chemokine release normalized to sham-treated islets.

After analysis of cell-depleted media collected postculture, the observations made with hypoxia-induced ROS could be verified. The lack of oxygen induced an approximately three-fold increase of chemokine release in sham-treated controls (*p* < 0.001 vs. pre-culture; Figure 7A–F). Although the magnitude of secretion varied enormously between the different chemokines assessed, the release followed a very similar pattern as shown in Figure 7A–F. In contrast, the presence of islet BMPs substantially reduced the chemokine production measured in sham-treated islets by approximately 50%. Remarkably, COL-4 had no statistically significant inhibitory effect on chemokine production in islets when compared with sham-treated islets. Moreover, significant differences were found comparing COL-4 with PLC (*p* < 0.001 vs. sham-treated) and the combination (*p* < 0.01, *p* < 0.001 vs. sham-treated), which showed the largest anti-inflammatory potency amongst all islet BMPs assessed. The enormous capacity of PLC to decrease chemokine production was also demonstrated by comparison with L-521 (*p* < 0.01; Figure 7A–F).

Figure 6 demonstrates that the correlation coefficients between TNF-α, playing a central role in the chemokine network, and other proinflammatory cytokines such as IL-8 (r = 0.74, *p* < 0.001; Figure 6B), IL-1β (r = 0.87, *p* < 0.001; Figure 6C) and MCP-1 (r = 0.73, *p* < 0.001; Figure 6D) were highly significant. In contrast, VEGF-A (r = 0.53, *p* < 0.01) and IL-6 (r = 0.48, *p* < 0.01), which are not included in Figure 6, showed a much weaker correlation with TNF-α.

**Figure 8:**
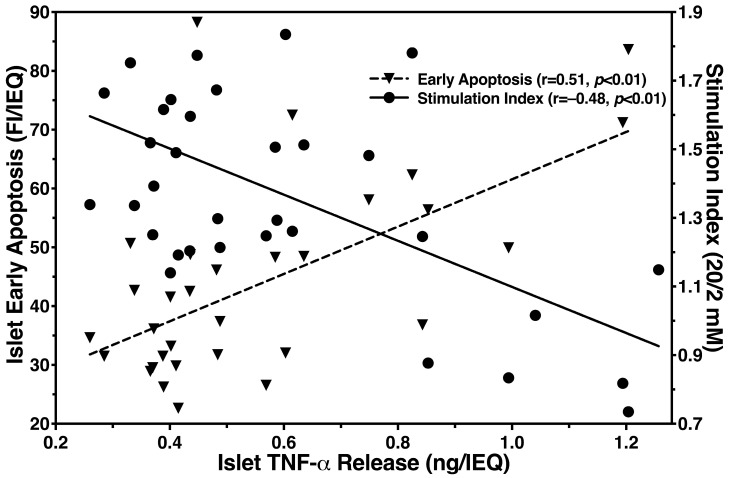
Effect of islet TNF-α release on early apoptosis (▼, dashed line, left y-axis) and glucose stimulation index (●, solid line, right y-axis). Each data point represents the intensity of Annexin-V staining or the glucose stimulation index in relation to the corresponding generation of TNF-α secretion. The correlation coefficient (r) was calculated using Spearman’s rank correlation after outlier identification (n = 32).

The detrimental effect of TNF-α on islet functional integrity is depicted in Figure 8. Whilst an inverse correlation of TNF-α was calculated for the GSI (r = −0.48, *p* < 0.01), the expression of phospatidylserine, a marker for early apoptosis correlated positively with TNF-α (r = 0.51, *p* < 0.01). A nearly identical correlation was found between TNF-α and late apoptosis (r = 0.50, *p* < 0.01) which is v in Figure 8.

The anti-inflammatory effect of islet BMPs was associated with a substantial reduction in early islet apoptosis which was significantly doubled in sham-treated islets when compared with islets pre-culture (*p* < 0.001; Figure 2E). Except for the combination group (NS vs. sham-treated, *p* < 0.01 vs. COL-4, PLC), all other islet BMPs were highly effective in inhibiting early apoptosis in comparison with the sham-treatment (*p* < 0.001 vs. COL-4, PLC; *p* < 0.01 vs. L-521). Moreover, the level of early apoptosis in islets treated with single BMPs was similar to islet early apoptosis measured pre-culture.

The expression of late apoptosis, characterized by the simultaneous expression of markers of apoptosis and necrosis, was only moderately enhanced in sham-treated islets when compared to pre-culture (*p* < 0.05, Figure 2F). Whilst the presence of COL-4, L-521 or PLC reduced late apoptosis to its pre-culture levels (NS vs. pre-culture; *p* < 0.01 vs. sham-treated), the combination of these three islet BMPs had a weaker effect on late apoptosis (NS vs. sham-treated, *p* < 0.05 vs. COL-4, PLC; Figure 2F).

## 4. Discussion

Throughout the isolation process, human islets are exposed to myriads of harmful variables severely affecting islet functional and morphological integrity. The central factors in this process are the interrupted supply of oxygen and nutrients, and the destruction of the natural pancreatic islet environment by collagenolytic enzymes and mechanical pancreas treatment [48]. Since the digestion of the ECM is an essential and inevitable key step of the islet isolation procedure, the reconstruction of the ECM may represent a significant option to improve islet survival prior to and after transplantation. Taking all these factors into account, it is obvious that the demand for a successful protective and regenerative islet treatment prior to transplantation is high.

Considering the tremendous complexity of the pancreatic ECM, and the endless quantity of different signals that are generated and harmonized by this structure to maintain tissue integrity, the decellularized pancreatic ECM represents the current gold standard for engineering a scaffold for isolated pancreatic islets [49]. Thus, it appears to be quite implausible that the functional potency of the whole pancreatic ECM can be replaced by individual isolated ECM components [7,50,51]. Nevertheless, before the use of decellularized ECM can be translated into clinical practice, major hurdles such as sterility, immunogenicity and standardization of the decellularized ECM have to be overcome [52]. In the meantime, the present study primarily used individual components of the islet BM that are already commercially available as sterile recombinant human proteins such as L-521 or PLC or that are extracted from human placenta like COL-IV, in order to limit the number of regulatory issues to be clarified for clinical application [51]. This strategy would also facilitate blending with encapsulation materials such as alginate [23,24].

The continuous presence of hypoxia during culture, implemented in the setting of our study to perpetuate the pro-inflammatory conditions as present during islet isolation and post-transplant, seems to be the major stimulus for the increased production of ROS and chemokines [53,54]. The present study revealed a tight correlation between nearly all chemokines assessed and outlined a chemokine network that comprises not only pro-inflammatory cytokines such as IL-1β, IL-6 and TNF-α but also protective ones like VEGF [55] or ambivalent chemokines such as IL-8 [56]. Our observations about the close relationship between different chemokines are in agreement with a previous study about the chemokine production of cultured human islets prior to transplantation [57]. In this complex network, TNF-α is the central element [58,59,60], whilst ROS are the dominant mediator of TNF-α-induced activities [54,61,62].

A continuous ROS overproduction cannot be compensated by the relatively low anti-oxidative enzyme expression in islets [63,64,65] leading to a substantial stimulation of TNF-α [62,66] which, in turn, boosts the mitochondrial generation of ROS [54]. As a consequence of this harmful loop, the excessive ROS overproduction can result in the damage of oxidized enzymes and structural proteins [67], induction of apoptosis [68,69,70,71] and human islet dysfunction in vitro as well as post-transplant [57,72,73,74,75]. In contrast, our study could not demonstrate a significant correlation of ROS with islet yield, viability, secretory capacity and early or late apoptosis. Our present data rather suggest that hypoxia in human islets results in a strong pro-apoptotic response and in a relatively mild increase of necrotic cell death. Vice versa, the protective capacity of the islet BMPs was significantly more potent in early apoptosis when compared with necrosis as measured by the FDA-PI assay.

The most relevant finding of our study is that PLC has the most powerful anti-inflammatory and anti-apoptotic capabilities amongst all islet BMPs tested. This was demonstrated by the strongest reduction in terms of ROS production and by the massive decrease of chemokine secretion. Whilst the chemokine release in sham-treated islets was at least triplicated when compared with islets pre-culture, PLC-treated islets secreted chemokines in quantities which were similar to the pre-culture baseline. On the other hand, PLC had only a relatively weak protective effect regarding islet yield and morphological integrity which was significantly more pronounced in COL-4. Apart from this property, COL-4 had only weak capabilities to reduce the production and secretion of the chemokines assessed. However, the observation that the protective feasibilities seem to vary between different islet BMPs clearly indicates that one single islet BMP cannot protect against the full spectrum of harmful factors associated with islet isolation and transplantation. Despite our finding that the combination of several islet BMPs is not as effective as single BMPs, the necessity to combine different islet BMPs to obtain a wide range of protection is still valid.

Integrins are essential mediators of ECM-derived signals into the cellular interior [10,76,77]. Previous studies localized several integrins on islets or beta cells isolated from different species and identified some metabolic pathways triggered by these integrins [11,12,16,18,33,34,78,79]. Nevertheless, since different BMPs can bind to the same integrins it is difficult to precisely attribute a defined effect of individual BMPs on certain anti-inflammatory and anti-apoptotic survival pathways [37,77,80]. In accordance with previous approaches, we could not detect any synergies between islet BMPs when added in combination [22,35,81,82] which may indicate a competitive ligation of different BMPs to the same integrins. The close cross-linking of the different chemokines, as shown in the present study, additionally aggravates the identification of specific effects of the islet BMPs tested. Further studies with competitive antibodies will be required in the future to identify the most relevant islet BMP-specific integrins [83]. This may also facilitate the definition of the ideal stoichiometric ratio between the most relevant components of the islet BM [23,84].

## 5. Conclusions

The present study demonstrated that PLC has the most potent anti-inflammatory capacity when compared with COL-4 and L-521, whilst COL-4 has the strongest potency to preserve islet yield. Combining COL-4, L-521 and PLC did not result in a further increase of islet protection against hypoxia-related impacts. From this study, we conclude that PLC is an effective anti-inflammatory and anti-apoptotic BMP candidate for islet encapsulation and transplantation but with a relatively low capacity to protect islet morphological integrity.

## Data Availability

Data are included within the article.
